# Lithium Impacts on the Amplitude and Period of the Molecular Circadian Clockwork

**DOI:** 10.1371/journal.pone.0033292

**Published:** 2012-03-12

**Authors:** Jian Li, Wei-Qun Lu, Stephen Beesley, Andrew S. I. Loudon, Qing-Jun Meng

**Affiliations:** 1 Faculty of Life Sciences, University of Manchester, Manchester, United Kingdom; 2 Faculty of Fisheries and Life Sciences, Shanghai Ocean University, Shanghai, China; Vanderbilt University, United States of America

## Abstract

Lithium salt has been widely used in treatment of Bipolar Disorder, a mental disturbance associated with circadian rhythm disruptions. Lithium mildly but consistently lengthens circadian period of behavioural rhythms in multiple organisms. To systematically address the impacts of lithium on circadian pacemaking and the underlying mechanisms, we measured locomotor activity in mice *in vivo* following chronic lithium treatment, and also tracked clock protein dynamics (PER2::Luciferase) *in vitro* in lithium-treated tissue slices/cells. Lithium lengthens period of both the locomotor activity rhythms, as well as the molecular oscillations in the suprachiasmatic nucleus, lung tissues and fibroblast cells. In addition, we also identified significantly elevated PER2::LUC expression and oscillation amplitude in both central and peripheral pacemakers. Elevation of PER2::LUC by lithium was not associated with changes in protein stabilities of PER2, but instead with increased transcription of *Per2* gene. Although lithium and GSK3 inhibition showed opposing effects on clock period, they acted in a similar fashion to up-regulate PER2 expression and oscillation amplitude. Collectively, our data have identified a novel amplitude-enhancing effect of lithium on the PER2 protein rhythms in the central and peripheral circadian clockwork, which may involve a GSK3-mediated signalling pathway. These findings may advance our understanding of the therapeutic actions of lithium in Bipolar Disorder or other psychiatric diseases that involve circadian rhythm disruptions.

## Introduction

Bipolar Disorder (BPD), also known as manic-depressive illness, is a mood disorder that affects 1–3% of the general population. Accumulating evidence supports the association of the disrupted circadian rhythms with the pathogenesis and manifestation of BPD [1]–[3]. For instance, during both the manic and the depression episodes, patients show profound disturbances in sleep cycles and hormonal secretion rhythms. For the last 60 years, lithium has been the mainstay treatment for BPD. Lithium lengthens the period of behavioral circadian rhythms in rodents and humans [4], as well as the circadian firing rate rhythms in dispersed SCN neurons [5]. However, the impacts of lithium on the dynamics of clock gene/protein rhythms in the SCN and peripheral tissues have not been critically investigated.

Circadian rhythms are generated by the cell autonomous endogenous circadian clocks. In mammals, the master circadian clock resides in the suprachiasmatic nuclei (SCN) of the hypothalamus. Output from the SCN synchronizes via multiple pathways peripheral oscillators in most body organs [6]–[8]. Within the pacemaker cells, operation of the circadian clock relies critically on the transcriptional/translational feedback loops. Circadian transcription is initiated by two bHLH-PAS domain-containing proteins CLOCK and BMAL1, which heterodimerize and activate in an E-box dependent manner the transcriptional repressors PERIOD (PER) and CRYPTOCHROME (CRY). Following a delay, PER and CRY proteins are rhythmically translocated to the nucleus to inhibit their own and other E-box regulated promoters. *Bmal1* is rhythmically regulated by two nuclear hormone receptors, which act as activator (RORα) or repressor (REV-ERBα) of transcription via common RORE elements on the *Bmal1* promoter [9], [10]. Glycogen synthase kinase 3β (GSK3β)-mediated phosphorylation has been implicated in the regulation of the stability and/or nuclear translocation of PER2, CRY2, CLOCK, REV-ERBα and BMAL1 [11]–[15].

As a competitive inhibitor of magnesium, lithium directly inhibits the ATP-magnesium-dependent catalytic activity of GSK3β [16], [17]. Lithium also indirectly inhibits GSK3β activity through enhanced phosphorylation of GSK3β at Ser9. Inhibition of GSK3β activity have been proposed as a key pathway mediating the effects of lithium on the circadian clocks [12], [18], [19]. However, other studies [20] have demonstrated an opposing period shortening effect upon GSK3β suppression in cultured mammalian cells, contrasting the period lengthening effects of lithium. Therefore, it has become pressing to understand whether the period lengthening is the major effect of lithium within circadian clockwork, and if so, what pathways are involved.

To address this, we performed wheel-running of mice treated chronically with lithium, and also monitored clock gene/protein dynamics in real-time following acute lithium treatment *in vitro*. We present the following findings, 1) Lithium treatment lengthens circadian period of behavioural rhythms, as well as the molecular pacemaking in the SCN and periphery. 2) Expression and oscillation amplitude of PER2::LUC in ectopic tissue explants (SCN and lung) and isolated cells are significantly augmented by lithium. 3) Increased PER2 expression in lithium-treated cells is associated with augmented *Per2* mRNA transcription, which can be phenocopied by a selective GSK3 inhibitor, but does not appear to involve the activity of PI3K/AKT. Our data therefore identified a novel effect of lithium treatment on the amplitude of PER2 protein rhythms, which may involve GSK3-mediated mechanisms.

## Materials and Methods

### Ethics statement

The mouse work described here was approved by the University Animal Ethical Review Group and conducted under a project licence (1986 Home Office Animal Procedures Act) granted by the UK Home Office.

### Animal maintenance and behavioral analysis

PER2::LUC mice were kindly provided by Professor J. Takahashi (the University of Texas Southwestern Medical Center at Dallas). In this knock-in mouse, endogenous PER2 protein is fused in-frame with a luciferase reporter, allowing real-time monitoring of the PER2 protein dynamics by bioluminescence recording [21]. To compare lithium responses in cells with different decay rate of PER2, WT and CK1ε^tau^ mice on a PER2::LUC background produced in this laboratory were used [22]. Only female mice were used in these studies. All mice were maintained at 20–22°C, on standard rodent breeder or maintenance chow. For wheel-running studies, 10–14 week old wild-type C57BL/6 mice were single-housed in cages equipped with running wheels and *ad libitum* food, contained within a light-tight chamber at constant temperature (°C) and humidity (% H_2_O). Mice were entrained to a 12 hour light (1000 lux) and 12 hour dark (LD) cycle for 7–14 days and released into constant darkness (DD) for ∼28 days. From this point, lithium (10 mM) was dissolved in drinking water, a dosage which previous reports show results in a brain concentration of about 1 mM in mice, close to the therapeutic blood level in man of 0.5–1 mM [4]. Saline was supplemented to adjust osmolality and avoid hyponatremia. Chronic lithium treatment lasted 28–35 days in DD. Running wheel activity was recorded and analyzed using ClockLab software as described previously [22].

### Reagents and antibodies

Lithium Chloride, dexamethasone, cycloheximide (CHX), AKT inhibitor (A6730) and collagenase IA were purchased from Sigma. PI3K inhibitor (LY294002) was purchased from Cell Signaling. GSK3α/β inhibitor (BIO, (2′*Z*,3′*E*)-6-Bromoindirubin-3′-oxime) [23], [24] was purchased from Calbiochem. Lithium Chloride was dissolved in water. A6730, LY294002 and BIO were dissolved in DMSO. The final concentrations of DMSO used were less than 0.01% in all experiments.

### Cell culture and preparation of organotypic tissue slices

Primary lung fibroblasts from WT or CK1ε^tau^ mice (both on the PER2::LUC background) were isolated as described previously [22]. Lungs were removed from adult mice killed by cervical dislocation, chopped and minced. Cells were then dissociated by means of shaking at 37°C for 2 hours in 100 u/ml of Collagenase IA. Rat1 cell lines with or without the stably transfected *Bmal1::luc* circadian reporters were used in real-time photon counting or Q-PCR studies [25].

SCN and Lung slices from WT PER2::LUC mice were prepared and cultured as described before [22]. Using a vibraslice (Integraslice 7550 MM; Campden, Loughborogh), 275 µm serial sections of brain (containing the SCN) or lung lobes were cut in 4°C HBSS. SCN was dissected out under a dissection microscope. Tissue slices were plated out onto Millicell 30 mm cell culture plate inserts (Millipore) prior to synchronization and photon counting.

### Bioluminescence recording and imaging

Real-time photon counting and bioluminescence imaging were performed using Photomultiplier tube assembly (PMT, H6240 MOD1, Hamamatsu Photonics) or a CCD camera as described previously [25], [26]. Confluent cells, SCN or lung slices in 35 mm dishes were synchronized by 100 nM dexamethasone for 1 hour before bioluminescence recording or imaging. Data were presented as photon counts per minute. Baseline correction was calculated using a 24-hour moving average, which removed the first 12 hours data. For the drug treatment experiments, 24 hours after synchronization, individual dishes with cells or tissue slices under PMT recording were treated with various drugs or vehicle control. The treatment agent was left continuously with the samples thereafter while the luminescence patterns were recorded. Period was analyzed by RAP software as described before [22], [27]. Amplitude was measured as the peak-trough difference 24–48 hrs after drug treatment, based on the baseline-corrected data.

### PER2::LUC decay assay

Real-time monitoring of PER2::LUC degradation was performed as described before [22]. Dexamethasone-synchronized fibroblasts or lung slices were treated with lithium for 48 hrs, followed by CHX (20 µg/ml). Bioluminescence data after CHX treatment were collected and analyzed by Prism (GraphPad Software) using one-phase exponential decay curve fitting. Half-lives were plotted as mean± SEM.

### TAQMAN Real-time quantitative PCR

Total RNA isolated from Rat1 cells were reverse transcribed and subjected to cDNA synthesis (TaqMan® Reverse Transcription Reagents, Applied Biosystems). The following primer pairs and probe for rat *Per2* were used (5′ to 3′): forward: GCAGGCTCACTGCCAGAACT; reverse:CAAGATGATTCTATTCCAGAAGCATT; Probe: AGCCCCAGCAAGTGATCGAGGACTAAG [28]. Rat housekeeping gene *rβ-actin* was used as an internal control. *rBeta-actin* forward: CGTGAAAAGATGACCCAGATCA; reverse: CACAGCCTGGATGGCTACGT; Probe: TTTGAGACCTTCAACACCCCAGCCA. The amplification and data collection was performed on an ABI 7300 (Applied Biosystems). Each sample was run in triplicate. The data was analysed using the 2^−ΔΔCT^ method [29].

### Statistical analysis

Statistical analysis was performed based on single experiments with several samples (biological replicates). Each experiment was repeated at least on 3 separate occasions, all repeats gave similar results. All data were presented as mean ± SEM, and compared by one way ANOVA. The only exceptions were the experiments using the AKT and PI3K inhibitors, which were based on single experiment without repeats.

## Results

### Lithium lengthens circadian period in behavioural rhythms

**Figure 1:**
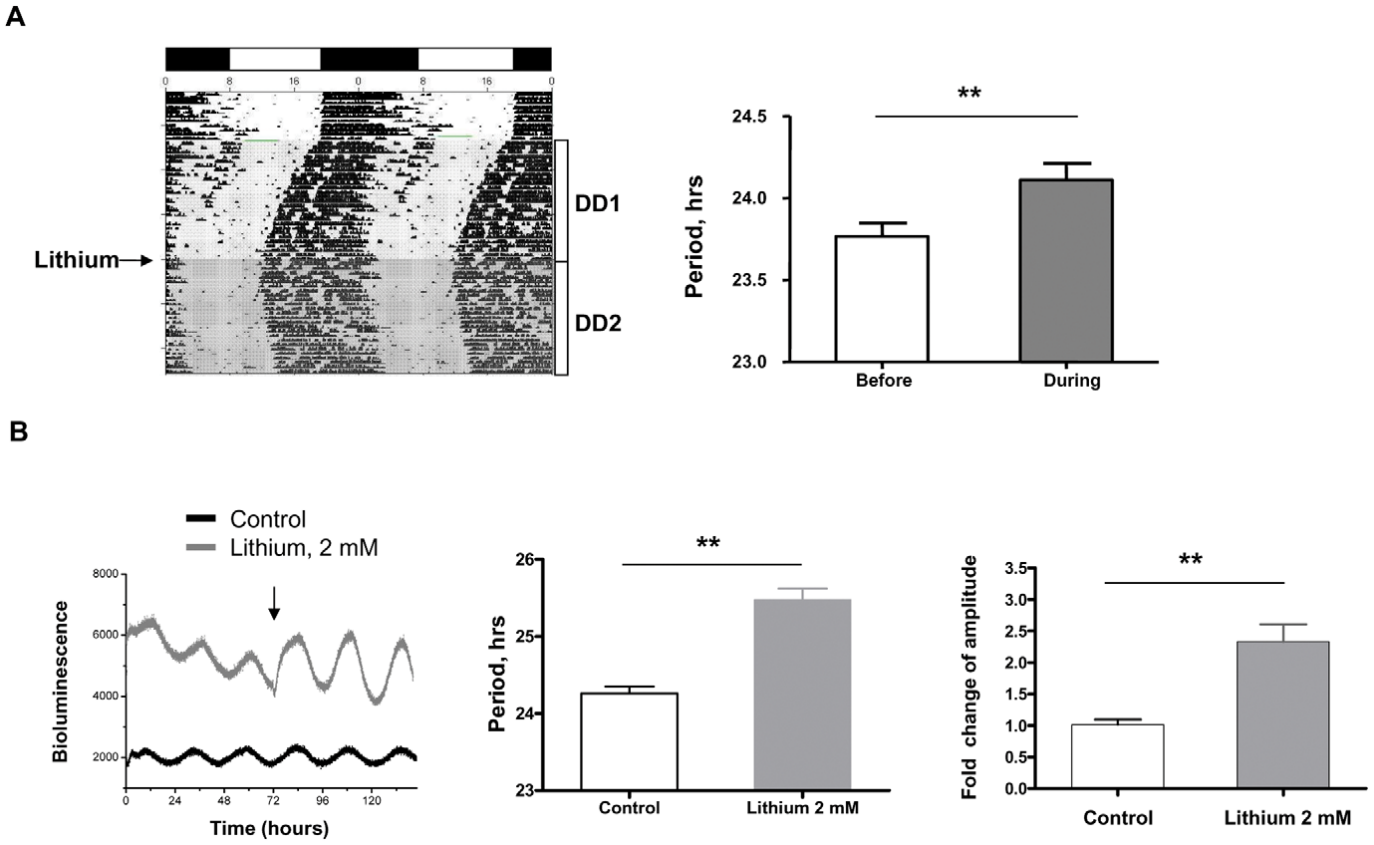
Lithium lengthens period for behavioral rhythms and alters molecular oscillations in the SCN. A, Representative behavioral rhythm and period analysis for WT C57BL/6 mice. Black arrow indicates initiation of lithium treatment (10 mM in drinking water). DD, constant darkness. Data were expressed as mean ± SEM; **, p<0.01, n = 6. B, Representative traces of PER2::LUC bioluminescence oscillations and analysis of period and amplitude in organotypic SCN slices recorded by PMT photon counting. Black arrow indicates time of lithium treatment. Untreated group was used as control. Data were expressed as mean ± SEM; **, p<0.01, n = 6.

Lithium is known to lengthen circadian free-running rhythms in a variety of species, including humans and rodents. By measuring the locomotor activity rhythms, we first confirmed the behavioural responses of wild type C57BL/6 mice following chronic treatment of lithium chloride in drinking water. During the vehicle treated period, mice wheel-run in constant darkness (DD) exhibited a periodicity of 23.77±0.08 hours, in line with the reported behavioural period length for C57BL/6 mice (Fig. 1A). Following lithium treatment, there was a mild but significant increase in circadian period (Fig. 1A, 24.11±0.10 hours, p<0.01, n = 6).

### Lithium impacts on clock period and PER2::LUC oscillation amplitude in the central and peripheral clockwork

Lithium treatment of dispersed SCN neurons lengthens the circadian period of firing rate rhythms in a dose-dependent manner [5]. To address whether lithium acts on the clock protein dynamics of the central pacemaker *in vitro*, we treated organotypic slices of SCN from the PER2::LUC mice with lithium. Administration of lithium at 2 mM led to significant lengthening of circadian period by ∼1.2 hours (p<0.01, n = 6, Fig. 1B), in agreement with the behavioural period lengthening effect. Interestingly, we also observed an elevation of PER2::LUC expression and increase of oscillation amplitude (2.45±0.86 fold vs. control, p<0.05, n = 6, Fig. 1B).

**Figure 2:**
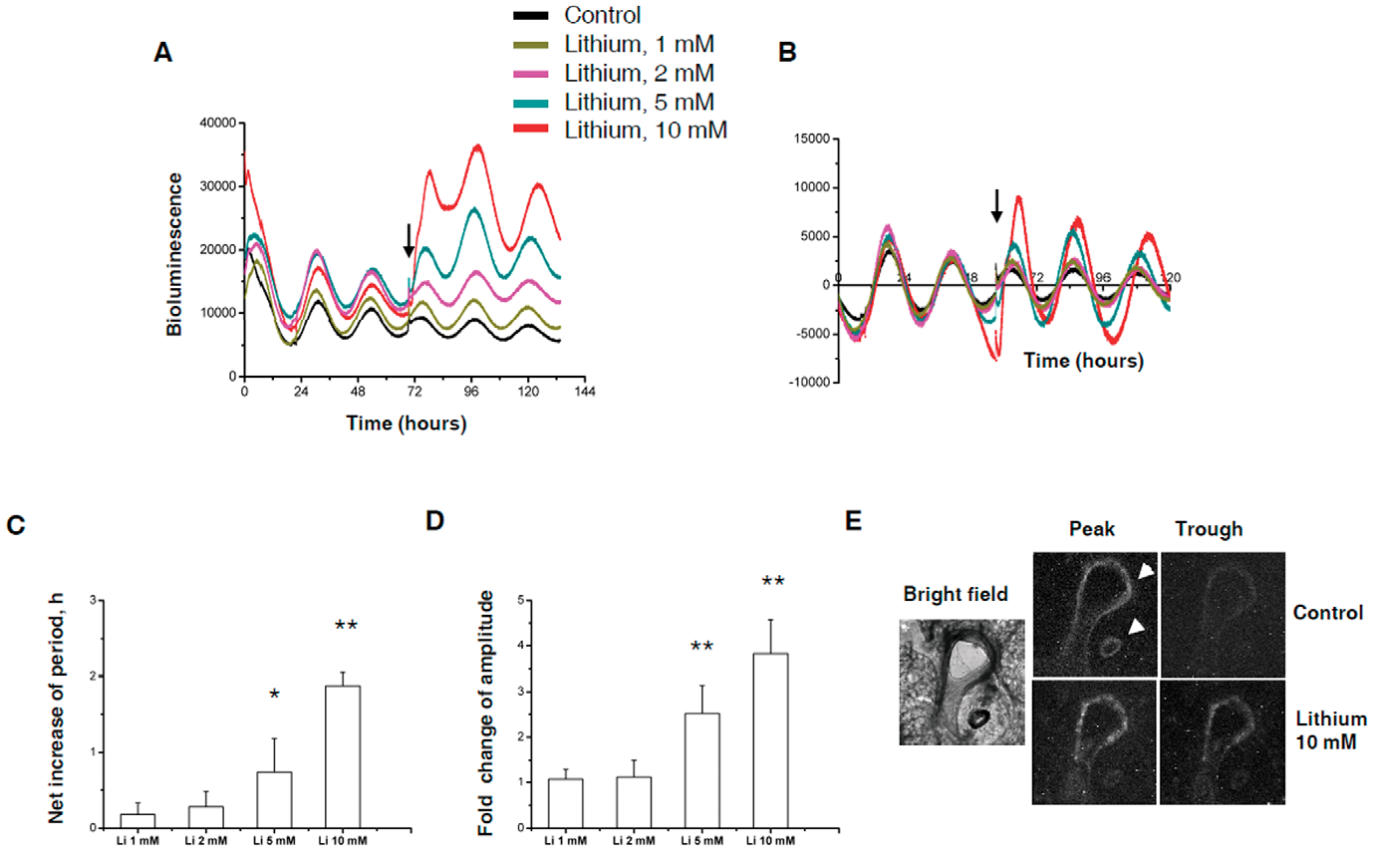
Lithium lengthens circadian period and enhances PER2::LUC expression in lung slices. A,B, Representative PER2::LUC traces from ectopic lung slices (A, raw data; B, baseline correction of raw data). Black arrow indicates time of lithium treatment. Untreated group was used as control. C,D, Period and amplitude analysis. The net period changes were analyzed by normalizing to the control slices and expressed as mean ± SEM. The amplitude changes were analyzed based on the first peak after treatment in the baseline corrected data. *, p<0.05; **, p<0.01, n = 5. E, Bright field and bioluminescence imaging of lung slices. Peak and trough of oscillations were determined based on the bioluminescent intensity of the CCD camera recording. White arrowheads indicate bronchiole and blood vessel.

To extend these studies to non-neural oscillators, we measured PER2::LUC bioluminescence rhythms in ectopic lung slices. Here, we observed clear dose-dependent period lengthening of PER2::LUC rhythms by lithium treatment, with ∼2 hour extension at 10 mM (p<0.01, n = 5). Similar to the SCN, there was a significant elevation of PER2::LUC expression, with 3.83±0.74 fold induction of amplitude following 10 mM lithium treatment (Fig. 2A–D). Further bioluminescence imaging using a CCD camera revealed that the elevated bioluminescence originated predominantly from the bronchioles and blood vessels (Fig. 2E), structures within the lung where the PER2 and CLOCK proteins are known to be predominantly expressed [30].

**Figure 3:**
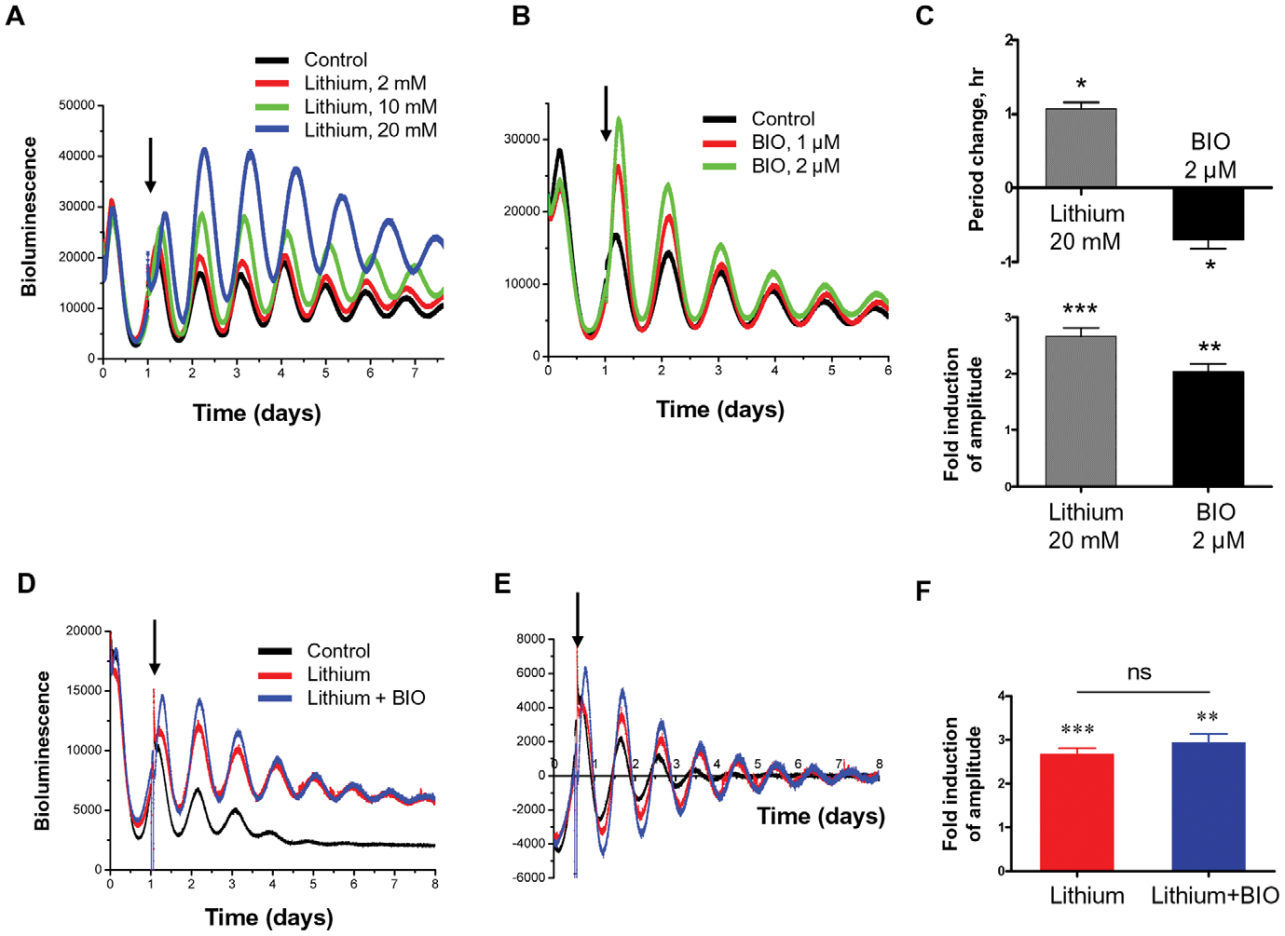
Effects of lithium and GSK3 inhibition on PER2::LUC expression and amplitude of oscillation. A,B, Representative traces of PER2::LUC rhythms from lung fibroblasts treated with increasing doses of lithium (A) or the GSK3 inhibitor BIO (B). Black arrow indicates time of treatment. Untreated group was used as control for lithium; DMSO was used as control for BIO. C, Period and amplitude analysis for PER2::LUC rhythms in lung fibroblasts following treatment with either lithium (20 mM), or BIO (2 µM). Mean ± SEM; *, p<0.05 vs. Control; **, p<0.01 vs. Control; ***, p<0.001 vs. Control; n = 4 for each group. D,E, Representative raw traces (D) and baseline corrected traces (E) of PER2::LUC rhythms from lung fibroblasts treated with lithium alone (20 mM), or in combination with BIO(2 µM). DMSO was used as vehicle control for BIO. F, Amplitude analysis for data in (D) and (E). **, p<0.01 vs. vehicle; ***, p<0.001 vs. vehicle; n = 4 for each group.

Cultured cells harbour self-sustained and cell-autonomous circadian clocks that resemble the molecular oscillations in the brain, therefore providing an amenable *in vitro* clock model to perform more detailed mechanistic studies [31], [32]. Similar responses to lithium were also observed in isolated lung fibroblasts (Fig. 3A,C). There were significant dose-dependent period lengthening effects (0.7±0.22 hr and 1.1±0.28 hr net increase at 10 and 20 mM, respectively, p<0.05, n = 4), associated with significantly increased amplitude of PER2::LUC oscillations (2.70±0.25 fold at 20 mM, p<0.001, n = 4, Fig. S1). In contrast to ectopic SCN tissues, there were no significant effects on either period or amplitude of PER2::LUC oscillation in lung slices or fibroblasts treated with lower doses (1 or 2 mM) of lithium, indicating likely tissue differences in sensitivity to lithium.

### The role of GSK3 and AKT in lithium-induced clock changes

Inhibition of GSK3β by lithium has been proposed as the underlying mechanism of the behavioural period-lengthening effect. To discriminate the contribution of GSK3β in the lithium-induced period and amplitude effects, we treated lung fibroblasts with a potent GSK3 inhibitor, BIO ((2′*Z*,3′*E*)-6-Bromoindirubin-3′-oxime). In contrast to the period lengthening effect of lithium, BIO significantly shortened circadian period in lung fibroblasts (by 0.7 hour at 2 µM, p<0.05, n = 4, Fig. 3B,C). However, similar to lithium, BIO significantly increased PER2::LUC expression and oscillation amplitude (∼2 fold amplitude change at 2 µM, Fig. S1). To explore the potential interactions between lithium and GSK3 inhibition, fibroblast cells were treated with lithium (20 mM), in the presence or absence of BIO (2 µM). No significant additive effects on amplitude of PER2 expression were observed compared to lithium treatment alone (lithium alone, 2.67±0.15 fold; combined, 2.90±0.34 fold, p>0.05, n = 4, Fig. 3D–F). These findings indicate that although GSK3 inhibition cannot explain the period lengthening effect of lithium, it may play a role in the increase of PER2::LUC amplitude upon lithium treatment.

PI3K-mediated AKT activation has also been shown as a target of lithium in cells [33]. To explore the potential involvement of AKT in lithium-induced PER2::LUC expression, lung fibroblasts were pretreated with an AKT inhibitor (A6730) at doses sufficient to suppress its kinase activity (5 fold IC_50_), followed by lithium treatment. Here, the lithium-induced PER2::LUC expression, increase of oscillation amplitude, and extension of period persisted, irrespective of the presence or absence of the AKT inhibitor (Fig. S2). Similar results were also found for a PI3K inhibitor (LY294002). Treatment of cells with LY294002 at a dose 7 fold of IC_50_ revealed no significant effects on lithium-induced clock changes (Fig. S3), thus indicating lack of involvement of the PI3K/AKT pathway in mediating Lithium actions on circadian clocks.

**Figure 4:**
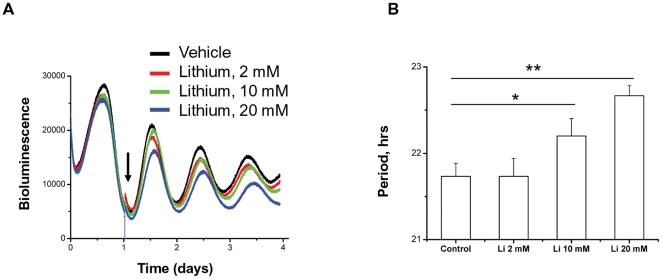
Lithium lengthens period of *Bmal1::luc* oscillation in Rat1 cells. A,B, Representative traces of PMT recording (A) and period analysis (B) for *Bmal1::luc* stable Rat1 cells treated with lithium. Mean ± SD; *, p<0.05 vs. Control; **, p<0.01 vs. Control; n = 4 for each group. Untreated group was used as control.

To investigate how lithium impacts on the dynamics of other clock genes, we next used Rat1 cells stably transfected with *Bmal1::luc* reporter [25]. Lithium lengthened period of *Bmal1::luc* transcriptional rhythms in a dose-dependent manner, but there was no significant elevation of *Bmal1*-driven bioluminescence level or oscillation amplitude (Fig. 4A, B), suggesting gene-specific effects of lithium within the molecular pacemaker.

### Lithium increases *Per2* mRNA levels and does not alter PER2 protein stability

**Figure 5:**
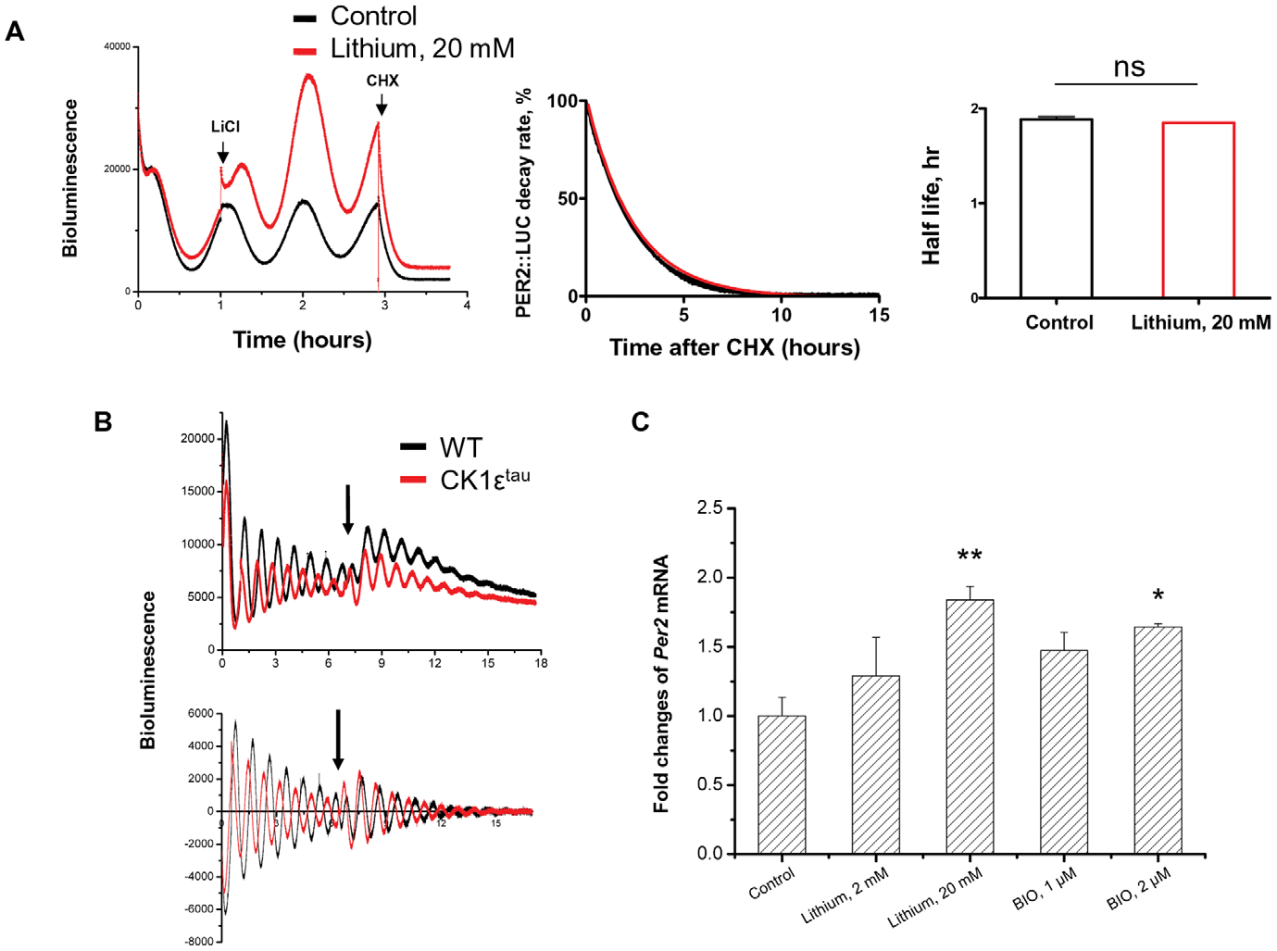
Lithium does not affect decay rate of PER2::LUC, but instead increases *Per2* transcription. A, Decay of PER2::LUC bioluminescence from lung fibroblasts was monitored by PMT recording. Fibroblasts were pretreated with lithium for 48 hrs, followed by treatment with cycloheximide (CHX, 20 µg/mL). Half-lives for PER2::LUC bioluminescence were analyzed by normalizing data to the peak level of expression (time 0 after CHX) and the minimum after 8 hours, and plotted as mean ± SEM. Untreated group was used as control. B, Lung fibroblasts isolated from WT and CK1ε^tau^ mice (on PER2::LUC background) were synchronized and subjected to PMT recording. Top panel, Raw traces; bottom panel, baseline corrected data. Black arrow indicates time of lithium treatment. C, *rPer2* mRNA level was quantified by Taqman Q-PCR in Rat1 fibroblasts synchronized with 100 nM dexamethasone. 24 hours later, cells were treated with different doses of either lithium or BIO for 12 hrs before total RNA collection. Data were normalized to *rβ-actin*. To calculate a relative change the average of the 3 replicate CT values for each sample was used. Mean ± SEM *, p<0.05; **, p<0.01; n = 4 for each group. Untreated group was used as control.

To address the possibility that lithium-induced PER2::LUC expression may be due to increased stability of PER2 protein, we measured the decay rate of PER2::LUC in lung fibroblasts following cycloheximide treatment (20 µg/ml) [22]. This revealed no significant effect of lithium on the half-life for PER2::LUC (1.89±0.03 hours for control, 1.85±0.01 hours for 20 mM lithium, p>0.05, n = 4, Fig. 5A). In cultured lung slices, a similar analysis revealed no significant change in half-life following 10 mM lithium treatment (2.15±0.12 hours for control, 2.11±0.08 hours for 10 mM lithium, p>0.05, n = 4). If lithium alters PER2 stability, we would predict that cells with different decay rate of PER2 may respond differently to lithium. To test this hypothesis, we used fibroblasts from the gain-of-function CK1ε^tau^ mutant mice as a model of accelerated PER2 degradation [22]. In common with studies of WT cells, an equivalent increase in the amplitude of PER2::LUC oscillation was observed in CK1ε^tau^ mutant fibroblasts (Fig. 5B,1.71±0.16 fold for WT vs.1.95±0.12 fold for CK1ε^tau^, p>0.05, n = 4), supporting our result of unaltered PER2 protein stability by lithium.

We next tested whether lithium may directly augment endogenous *Per2* transcription, leading to elevated PER2 protein expression. This was achieved by using quantitative RT-PCR for *Per2* mRNA in dexamethasone -synchronized Rat1 fibroblast cells. Upon lithium treatment, there was a clear dose-dependent increase of *Per2* mRNA level (∼2 fold at 20 mM, P<0.01, n = 4, Fig. 5C). Interestingly, BIO also significantly enhanced *Per2* mRNA level (∼1.7 fold at 2 µM, P<0.05, n = 4, Fig. 5C), consistent with our earlier observations of enhanced PER2::LUC expression by either lithium or BIO treatment. These results therefore suggest that lithium elicits induction of *Per2* transcription, but does not alter the degradation of PER2 protein in cells.

## Discussion

Lithium salt is considered to be one of the most effective drug treatments for long term mood stabilization in BPD patients. However, there is also large inter-individual variability in responses to lithium, and it can cause serious side effects. It is therefore important to understand the pharmacological actions and molecular targets. There is compelling evidence that BPD is associated with disruptions in circadian rhythms [2], [34], [35], and lithium has been shown to robustly lengthen behavioral circadian period in many species [4]. Consistent with earlier reports, our results demonstrate that lithium decelerates circadian behavioural rhythms and molecular pacemaking in the brain and peripheral tissues and cells. However, it is unclear whether lithium has other effects on the molecular circadian clockwork in addition to the altered circadian clock period.

The real-time recording of PER2::LUC rhythms in tissues/cells allowed us to systematically evaluate the effects of lithium on the circadian clockwork. In addition to the well-established period lengthening effect, we show here that the oscillation amplitude of the PER2 protein rhythms in both central (SCN) and peripheral clocks (lung and lung fibroblasts) is significantly augmented by lithium. There are at least three possible explanations. First, lithium might act as a synchronizing/resetting factor for the cellular clocks. This is unlikely, since lithium treatment in the Rat1 fibroblast cells did not synchronize/reset the circadian transcriptional cycles for *Bmal1::luc*. Secondly, lithium may reduce the degradation rate of PER2 proteins, leading to PER2::LUC accumulation, as GSK3β-mediated phosphorylation has been implicated in the regulation of the stability of REV-ERBα [12] and BMAL1 [13] through proteasomal degradation. This appeared not to be the case, as PER2 turnover, as inferred from changes in PER2::LUC decay rate, was unaltered following lithium treatment. Finally, lithium may regulate *Per2* at transcriptional or post-transcriptional level. This hypothesis is supported by our data, and by earlier reports which showed that lithium treatment selectively increased *Per2* mRNA levels in serum-shocked NIH 3T3 cells [19], [36]. Further studies are required to understand whether and how increased transcription of *Per2* leads to enhanced amplitude of PER2::LUC rhythms, as well as to explore the additional post-transcriptional or post-translational mechanisms.

Clock protein REV-ERBα has been proposed as a potential target for GSK3β and lithium [12]. These observations in HEK 293T cells provide a potential functional explanation for the actions of lithium on the circadian clockwork. However, our results using a *Bmal1::luc* reporter cell line that has RORE elements in the *Bmal1* promoter did not show significant alteration of *Bmal1::luc* bioluminescence upon lithium treatment. Consistent with our observations, studies using Northern blot analysis over a time course of 48 hours revealed no significant increase of endogenous *Bmal1* mRNA in NIH 3T3 cells upon lithium treatment [19]. Use of different cell line models may account for the discrepancies observed. Therefore, further studies are required to elucidate the contribution of REV-ERBα to lithium-induced clock changes in the tissue/cell models used in this study.

Our studies show that both lithium and GSK3 inhibition enhance PER2::LUC expression. An increase in amplitude by lithium independent of GSK3 inhibition would predict that combined treatment of lithium and GSK3 inhibitor should have additive effects. This hypothesis is not supported by our data, since fibroblast cells treated with both BIO and lithium demonstrated no further significant additive effects on amplitude of PER2 expression, indicating that inhibition of GSK3 may be the causal mechanism for induction of PER2 protein rhythms by lithium. The period effect, on the other hand, may involve additional mechanisms, as also suggested by Hirota et al [20].

GSK3β and the *Drosophila* ortholog, SHAGGY, have been shown to directly interact with and phosphorylate mPER2 and dPER protein, leading to nuclear translocation [15], [19]. Thus, delayed nuclear entry upon lithium treatment has been proposed as one potential mechanism for lithium-induced period lengthening. Within the molecular oscillator, PER2 acts as the negative regulator which suppresses transactivation of E-box regulated genes, including its own transcription. Therefore, it is conceivable that inhibition of GSK3β by lithium or BIO may block nuclear entry of PER2 proteins, hence relieving its negative repression on *Per2* transcription. This may consequently lead to the induction of *Per2* and high amplitude oscillation of PER2 proteins. Interestingly, recent studies using primary skin fibroblasts isolated from BPD patients and healthy controls failed to detect any period differences, but demonstrated significantly reduced oscillation amplitude of several clock genes, accompanied by reduced phosphorylation of GSK3β (hence enhanced GSK3β activity) [37].

One limitation of the current study is that in peripheral tissues, the concentration of lithium we used (∼10 mM for lung tissues and ∼20 mM for cells) were well above the therapeutic range of serum levels (0.5–1 mM). Similar doses have been used in other studies to reveal the effects of lithium on circadian clocks [12], [19], [20], [36]. Another limitation of this study is that we only addressed the potential roles of GSK3 and PI3K/AKT pathways in mediating lithium actions on the circadian clockwork. In fact, lithium in cells may trigger multiple distinct pathways. In addition to GSK3 and PI3K/AKT pathways explored here, there will be other known or yet to be identified targets that also impact on the circadian clockwork. Therefore, further investigation is warranted to identify additional mechanisms.

Collectively, our data demonstrate increased expression and oscillation amplitude of PER2::LUC rhythms by lithium in the SCN and peripheral tissues/cells, associated with decelerated behavioural and molecular pacemaking. Although the period effect of lithium may involve additional mechanisms, GSK3 inhibition phenocopies the increased *Per2* mRNA levels and enhanced amplitude of PER2::LUC rhythms. Our findings may shed new light on the understanding of the cellular and molecular mechanisms underlying therapeutic actions of lithium in Bipolar Disorder, and suggest that specific targeting of GSK3β in particular may offer novel treatment solutions for BPD patients.

## Supporting Information

Figure S1 **Amplitude effects of lithium and BIO in WT PER2::LUC fibroblast cells.** Representative traces of PMT recording to highlight the enhanced amplitude of oscillation by lithium (A) or BIO (B). Data were subjected to baseline correction which removed the first 12 hours data. Black arrow indicates time of lithium or BIO treatment.
(TIF)

Figure S2 **Lithium-induced PER2::LUC expression does not appear to involve AKT activity.** Representative traces of PER2::LUC rhythms from lung fibroblasts. Cells were first treated with the AKT inhibitor (A6730) as indicated by the arrow. After 1 hr, cells were treated with lithium (20 mM). A, raw data; B, baseline correction of raw data. Data are presented from a single experiment.
(TIF)

Figure S3 **Lithium-induced PER2::LUC expression does not appear to involve PI3K activity.** Representative traces of PER2::LUC rhythms from lung fibroblasts. Cells were first treated with the PI3K inhibitor (LY294002) as indicated by the arrow. After 1 hr, cells were treated with lithium (20 mM). A, raw data; B, baseline correction of raw data. Data are presented from a single experiment.
(TIF)
