# Supplementary figures and images for: Lithium Impacts on the Amplitude and Period of the Molecular Circadian Clockwork
Source: PLoS One. 2012 Mar 12;7(3):e33292. doi: 10.1371/journal.pone.0033292 (PMC3299767; doi:10.1371/journal.pone.0033292)

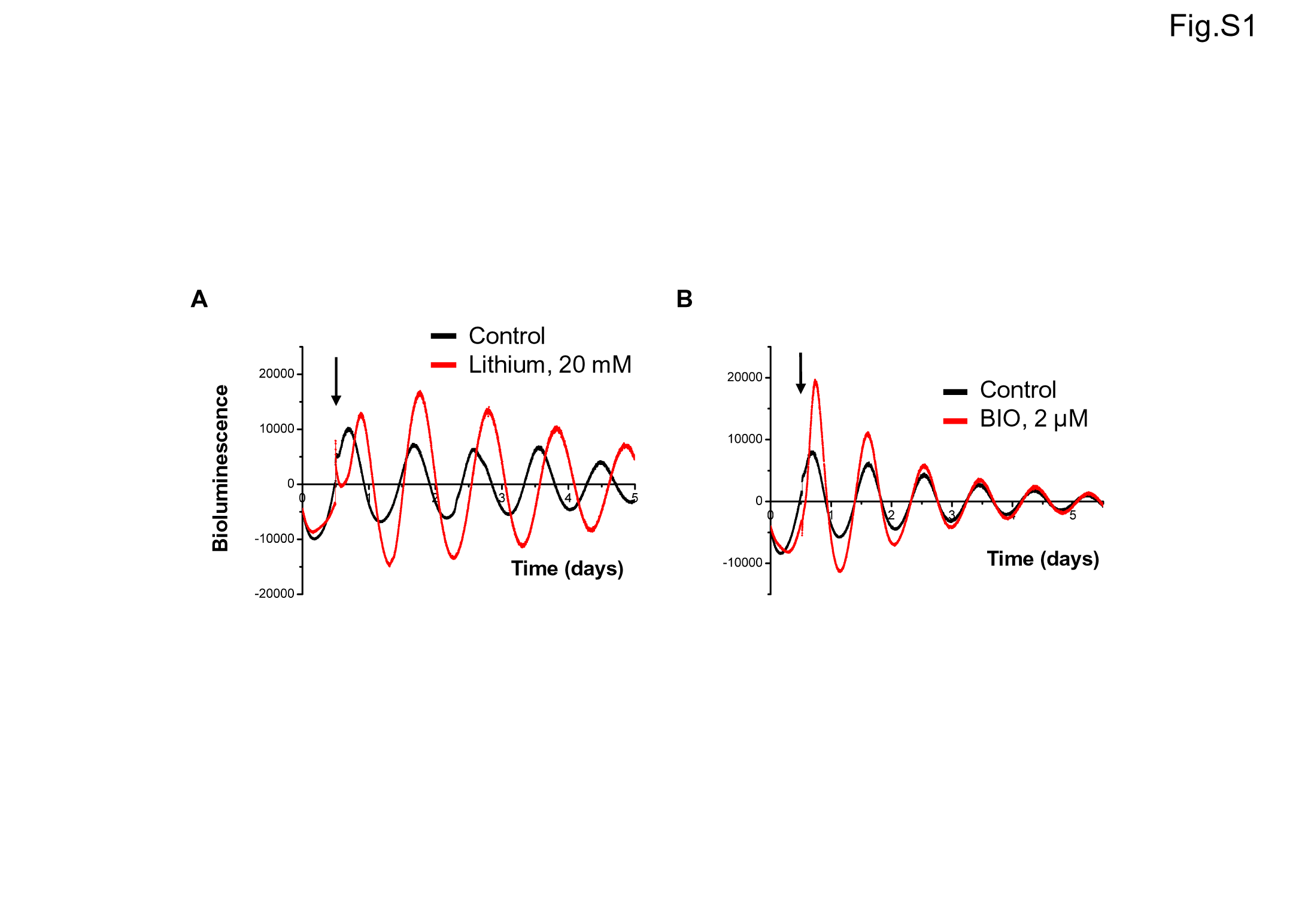

Supplement: Figure S1 — Amplitude effects of lithium and BIO in WT PER2::LUC fibroblast cells. Representative traces of PMT recording to highlight the enhanced amplitude of oscillation by lithium (A) or BIO (B). Data were subjected to baseline correction which removed the first 12 hours data. Black arrow indicates time of lithium or BIO treatment. (TIF) [file pone.0033292.s001.tif]

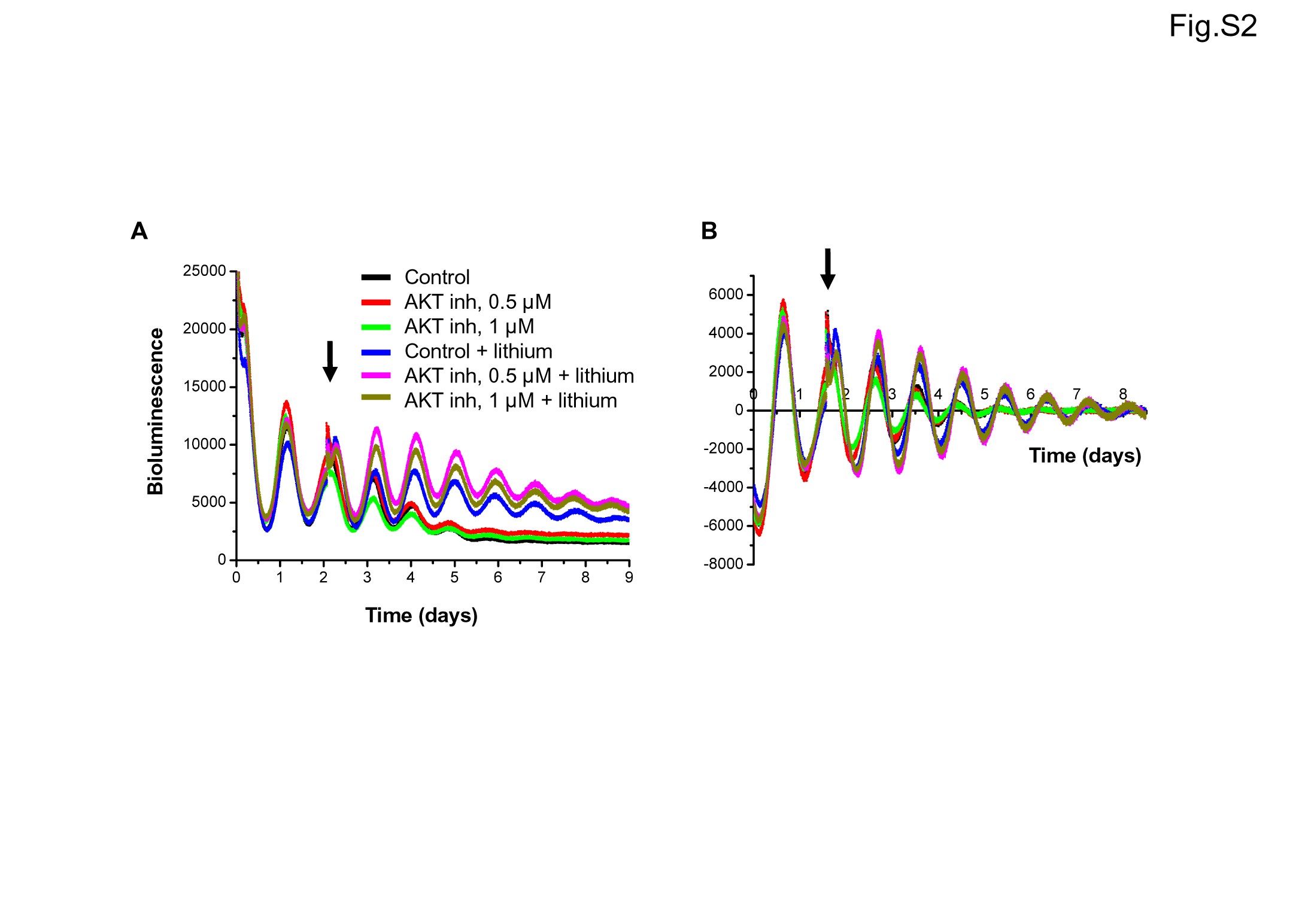

Supplement: Figure S2 — Lithium-induced PER2::LUC expression does not appear to involve AKT activity. Representative traces of PER2::LUC rhythms from lung fibroblasts. Cells were first treated with the AKT inhibitor (A6730) as indicated by the arrow. After 1 hr, cells were treated with lithium (20 mM). A, raw data; B, baseline correction of raw data. Data are presented from a single experiment. (TIF) [file pone.0033292.s002.tif]

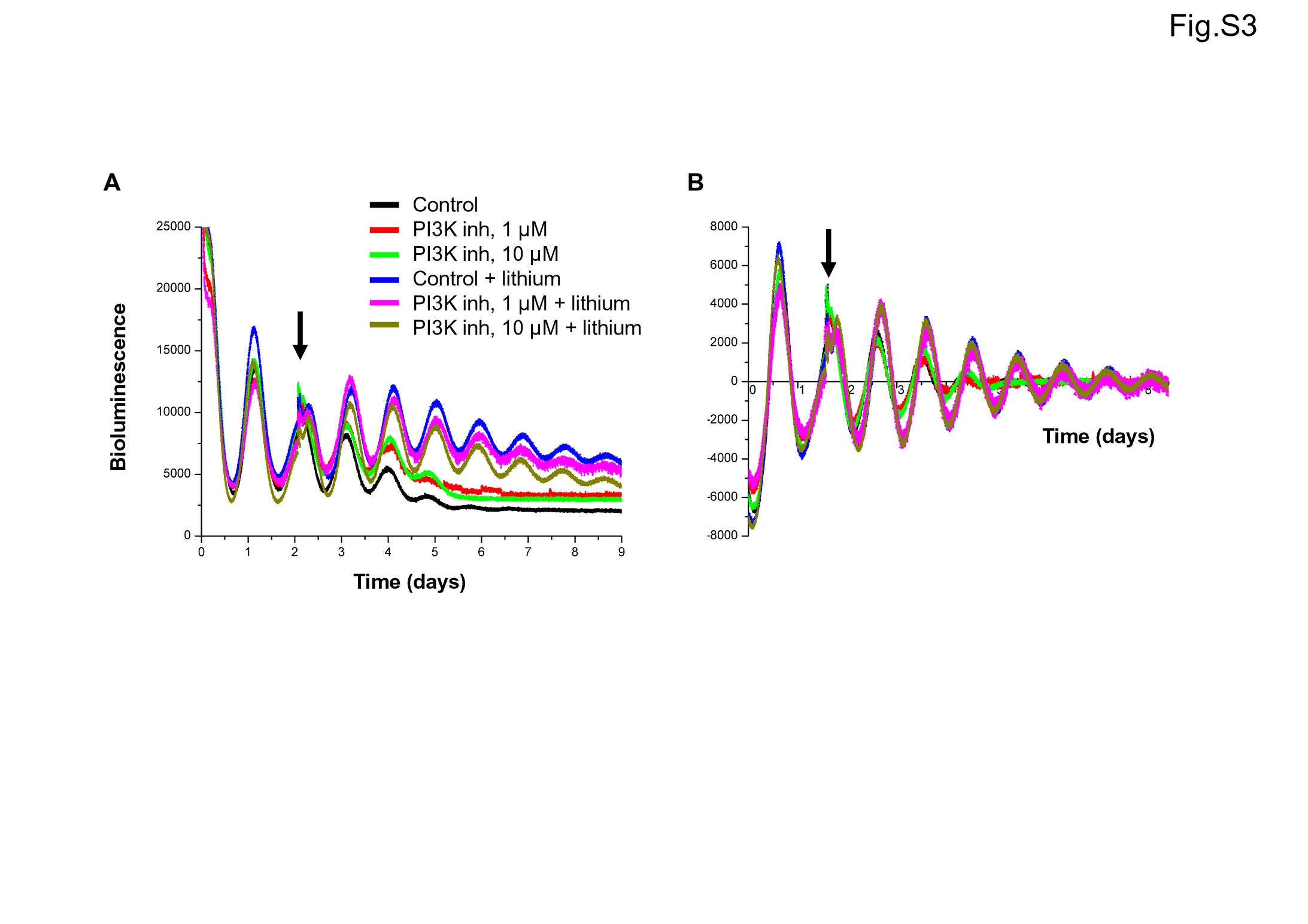

Supplement: Figure S3 — Lithium-induced PER2::LUC expression does not appear to involve PI3K activity. Representative traces of PER2::LUC rhythms from lung fibroblasts. Cells were first treated with the PI3K inhibitor (LY294002) as indicated by the arrow. After 1 hr, cells were treated with lithium (20 mM). A, raw data; B, baseline correction of raw data. Data are presented from a single experiment. (TIF) [file pone.0033292.s003.tif]
